# Adaptive growth strategies of *Quercus dentata* to drought and nitrogen enrichment: a physiological and biochemical perspective

**DOI:** 10.3389/fpls.2024.1479563

**Published:** 2024-11-22

**Authors:** Zipeng Zhao, Bing Xie, Xiaona Wang, Qi Wang, Chang Guo, Fang Zhang, Hongru Wang, Ruijie Zhang, Chen Zhang

**Affiliations:** College of Landscape Architecture and Tourism, Hebei Agricultural University, Baoding, China

**Keywords:** nitrogen deposition, water stress, soil enzyme activity, physiology, nutrient uptake

## Abstract

Nitrogen deposition and drought significantly influence plant growth and soil physicochemical properties. This study investigates the effects of nitrogen deposition and water stress on the growth and physiological responses of *Quercus dentata*, and how these factors interact to influence the overall productivity. Two-year-old potted seedlings were selected to simulate nitrogen deposition and water stress. Nitrogen was applied at rates of 0 kg·ha^-1^·year^-1^ (N0) and 150 kg·ha^-1^·year^-1^ (N150). The levels of water stress corresponded to 80% (W80), 50% (W50), and 20% (W20) of soil saturation moisture content. High nitrogen (N150) significantly increased stem elongation and stem diameter by enhancing photosynthetic parameters, including *P*_n_ (W80) and *G*_s_ (W50), and maintained higher water use efficiency. Under drought conditions, nitrogen enhanced leaf water content, stabilized electrical conductivity, regulated antioxidant enzyme activity, and increased the accumulation of proline. However, under severe drought, nitrogen did not significantly improve biomass, highlighting the critical role of water availability. Additionally, increased nitrogen levels enhanced soil enzyme activity, facilitated the uptake of crucial nutrients like K and Zn. Mantel tests indicated significant correlations between soil enzyme activity, water use efficiency, and leaf Fe content, suggesting that nitrogen deposition altered nutrient uptake strategies in *Q. dentata* to sustain normal photosynthetic capacity under water stress. This study demonstrates that nitrogen deposition substantially enhances the growth and physiological resilience of *Q. dentata* under W50 by optimizing photosynthetic efficiency, water use efficiency, and nutrient uptake. However, the efficacy of nitrogen is highly dependent on water availability, highlighting the necessity of integrated nutrient and water management for plant growth.

## 1 Introduction

The rate of nitrogen (N) deposition has markedly increased due to heightened anthropogenic activities, particularly the combustion of fossil fuels, presenting substantial ecological challenges (Hu et al., 2019; Gao et al., 2020; Zhang et al., 2021b). Arid and semi-arid regions, which comprise approximately 40% of the Earth’s terrestrial surface, are particularly vulnerable to changes induced by N deposition (Berdugo et al., 2020; Hu et al., 2021). These alterations in precipitation patterns, compounded by increased N deposition, are significantly influencing plant growth, community structure, and biogeochemical cycles within these ecosystems (Abid et al., 2016; Liu et al., 2017).

In adaptation to drought, plants have developed sophisticated physiological strategies to optimize water utilization. These adaptations include enhancing root development to improve nutrient and water absorption (Reich et al., 2014; Chen et al., 2019) and increasing water use efficiency (WUE) through reductions in stomatal conductance (*G*_s_) and transpiration rate (*T*_r_) (Roddy et al., 2018; Bourbia et al., 2020; Li et al., 2023a). Concurrently, plants must adjust to the cellular alterations triggered by drought stress. This involves strengthening antioxidant defenses to counteract damage from reactive oxygen species, employing enzymatic systems such as peroxidase (POD), superoxide dismutase (SOD), and catalase (CAT), which exhibit varied activity levels under drought conditions (Xiong et al., 2022). Additionally, plants accumulate soluble proteins (SP), soluble sugars (SS), and proline (Pro) for osmotic regulation, facilitating cellular adaptation to water scarcity (Daoudi et al., 2018).

The interaction between N deposition and drought conditions often results in multifaceted impacts that cannot be fully understood by considering their individual effects alone. Research has shown that N supplementation can alleviate the adverse effects of drought on plants, especially under conditions of soil water scarcity (Li et al., 2023b). For example, N-enhanced processes such as increased *G*_s_ facilitate greater gas exchange rates, which contribute to the accumulation of photosynthetic products and improve both the net photosynthetic rate (*P*_n_) and WUE (Dulamsuren and Hauck, 2021; Zhang et al., 2021a; Wang et al., 2024). While excessive N may initially stimulate plant growth and biomass, it can lead to rapid soil moisture depletion, thereby intensifying the effects of drought (Zhang et al., 2015). Additionally, N deposition can modify soil nutrient dynamics, potentially increasing the availability of certain elements such as iron (Fe) and zinc (Zn) in plant tissues (Płaza and Rzążewska, 2021). However, prolonged high levels of N deposition have been linked to soil acidification, reducing the bioavailability of essential nutrients like calcium (Ca), potassium (K), magnesium (Mg), and phosphorus (P) (de Jong et al., 2022). Nitrogen deposition also affects soil microbial activity, which influences nutrient cycling and decomposition processes. It can suppress biological N fixation, enhance soil nitrification and denitrification, and decrease the activity of N-fixing microorganisms, ultimately affecting overall soil nutrient availability (Zhou et al., 2018; Tafazoli et al., 2019). Moreover, N can alter microbial-mediated processes vital for the mineralization of organic P and the dissolution of inorganic P, crucial for maintaining soil P availability (Chhabra et al., 2013; Shi et al., 2022; Xu et al., 2022). Concurrently, drought conditions can reduce WUE, decrease *T*_r_, impair root absorption capabilities, and hinder nutrient transport. Additionally, soil water deficits can limit nutrient mineralization, further complicating nutrient uptake by plants (Manzoni et al., 2012).

*Quercus dentata*, a notable species within the Fagaceae family, is extensively distributed throughout Asia, especially in China. This oak species is recognized for its robust adaptation to poor soils and challenging drought conditions. It plays a crucial role in forest ecosystems, primarily due to its effective carbon sequestration capabilities (Lockwood et al., 2023). Despite its ecological significance, the interactive effects of drought and N deposition on *Q. dentata*, particularly under conditions characterized by abundant N and different soil moisture, remain insufficiently understood.

This study assesses the physiological responses of *Q. dentate* seedlings to varying levels of drought stress and N addition, examining their combined effects on gas exchange, biomass, nutrient content, and other physiological indicators. The primary objective is to elucidate how N supplementation influences drought tolerance mechanisms, with a specific focus on changes in physiological attributes and nutrient uptake strategies. Our hypotheses propose that: (1) nitrogen deposition counteracts the reduction in photosynthetic efficiency caused by drought, thereby supporting energy supply and biomass accumulation; (2) nitrogen deposition enhances nutrient utilization effectiveness, thereby helping to mitigate the impacts of drought; and (3) the interaction between drought stress and N deposition results in modified nutrient uptake and allocation strategies within the plants. Through exploring these interactions, the study aims to reveal the coping mechanisms employed by *Q. dentata* under the dual stresses of drought and N deposition. The findings are intended to provide valuable insights into strategies that enhance the growth and effective utilization of *Q. dentata*, thereby supporting ecosystem resilience and forestry management in response to global climate change.

## 2 Materials and method

### 2.1 Experimental site

The study was conducted at the experimental facility located at the Western Campus of Hebei Agricultural University, Baoding, Hebei Province, China (38°86′N, 115°48′E). This site is characterized by a temperate continental monsoon climate, with an average annual temperature of 13.4°C and an average annual precipitation of 498.9 mm, mostly occurring during the summer months due to monsoonal influence. The region benefits from a long frost-free period, averaging 211 days, facilitating extended growing seasons. Additionally, the area receives approximately 2513 hours of sunshine annually, ensuring ample solar exposure essential for plant growth. The mean annual temperature approximates 13.4°C, with a recorded maximum of 30°C and a minimum of 11°C observed during the sampling period.

### 2.2 Experimental design

This study was designed to evaluate the effects of simulated nitrogen (N) deposition and water stress on *Quercus dentata* seedlings. We used two-year-old uniformly grown seedlings, which were potted in containers measuring 31 cm in height and 28 cm in diameter, filled with a soil mixture composed of loam, sand, and peat in a 4:2:1 volumetric ratio. The soil’s electrical conductivity (EC) was recorded at 0.5 mS/cm, with a saturation moisture content was approximately 37.1%.

Nitrogen treatments were applied at two levels: 0 kg·ha^-1^·year^-1^ (control, N0) and 150 kg·ha^-1^·year^-1^ (high nitrogen, N150). Water stress was implemented at three levels of soil saturation moisture content: 80% (high moisture), 50% (moderate drought), and 20% (severe drought), creating a total of six treatment combinations (i.e., N0W80, N0W50, N0W20, N150W80, N150W50, N150W20). Nitrogen was supplied bi-weekly in the form of a urea [(NH_2_)_2_CO] solution, diluted 0.25g urea in 0.5 L of distilled water, with a comparable volume of distilled water applied to control pots, a total of eight times.

Relative water content (RWC) was consistently monitored using a portable meter, with 12cm probe (Field Scout TDR300, Spectrum Technologies, Chicago, USA).The water treatments were maintained throughout the entire growing season by measuring the RWC every three days. To eliminate the interference of direct rainfall, all plants were sheltered, and each pot was equipped with a collection tray to minimize water and nutrient loss. A completely randomized design was employed, featuring 6 experimental plots, totaling 144 trees, with random sampling to be conducted at the end of the growing season.

### 2.3 Photosynthetic indices

Approximately in the end of the growing season, we select a clear morning (from 9:00 to 10:00 am) to measure photosynthetic parameters, including net photosynthetic rate (*P*_n_), stomatal conductance (*G*_s_), intercellular CO_2_ concentration (*C*_i_), and transpiration rate (*T*_r_). These measurements were conducted using a Li-6400XT Portable Photosynthesis System (LI-COR, USA). To ensure uniform environmental conditions during the assessments, a constant light intensity of 900 µmol·m^-2^·s^-1^ was maintained using LED lamps (with 6 cm^2^ leaf chamber). For each treatment, 4 plants were randomly selected, and measurements were taken from one mature, healthy leaf in the upper-middle part of the plants. The measurements were conducted at 60% RH, an air temperature of 25°C, and a CO_2_ concentration of 400 µmol·mol^-1^.

Leaf samples were collected at the same time as the photosynthesis measurements, with four replicates for each treatment. Chlorophyll content was quantified using the ethanol extraction method. Briefly fresh leaf samples were collected immersed in 95% ethanol, and the absorbance of the resulting solution was measured at 645 nm, 663 nm, and 470 nm using a microplate reader (SpectraMax Plus 384 Molecular Devices, Sunnyvale, USA).

### 2.4 Plant sampling and measurement parameters

*Sample Collection*: At the end of the four-month experimental duration, four representative seedlings from each treatment were selected for detailed destructive analysis. These seedlings were carefully washed, immediately labeled, sealed in plastic bags, placed on ice, and transported to the laboratory for subsequent analyses.

*Plant Growth Indices*: Measurements of plant growth like height and stem diameter were recorded at both the beginning and end of the experimental period. Fresh and dry weights of the shoot and root biomass were documented. The leaf water content was determined using the leaf dry weight method (Zhang et al., 2024). When measuring the dry weight of plant tissues, various parts of the plant are first placed in an oven at 115°C for a half-hour to terminate biological processes, followed by drying at 75°C until a constant weight is achieved.

*Physiological Assessments*: Relative electrical Conductivity (REC) of plant leaf was measured using a glass electrode (Meng et al., 2015). Malondialdehyde (MDA) content, indicative of lipid peroxidation, its levels and free proline (Pro) concentrations serving as both a stress indicator and osmoprotectant were assessed using the thiobarbituric acid method and the acid ninhydrin method, respectively (Schweet, 1954; Nazari et al., 2012). Soluble sugars (SS) and proteins (SP) were quantified using the phenol-sulfuric acid method and the Coomassie brilliant blue G-250 colorimetric method, respectively (Bradford, 1976; Buysse and Merckx, 1993). Superoxide dismutase (SOD) activity was evaluated using the nitroblue tetrazolium (NBT) method (Beyer and Fridovich, 1987). Peroxidase (POD) activity was determined by measuring absorbance at 470 nm with slight modifications. Catalase (CAT) activity was measured through an improved potassium permanganate titration method (Yu et al., 2017). Water use efficiency (WUE) was calculated from the ratio of net photosynthetic rate (*P*_n_) to transpiration rate (*T*_r_).

*Nutrient Analysis*: Plant samples were segregated into main roots, fine roots, stems, twigs, and leaves for chemical analysis. Samples were ground, digested at 230°C using a wet digestion technique with H_2_SO_4_, and treated with H_2_O_2_ during digestion (Hou et al., 2021). Elemental analysis for potassium (K), calcium (Ca), magnesium (Mg), iron (Fe), manganese (Mn), copper (Cu), zinc (Zn), and boron (B) was conducted using inductively coupled plasma spectrometry (Prodigy, Spec, Leeman Labs Inc., USA). Total phosphorus (TP) was determined by the molybdenum blue reaction colorimetric method, and total nitrogen (TN) contents were analyzed using an automated chemical analyzer (Smartchem 600, AMS-Alliance, Italy)

### 2.5 Soil sampling and measurement parameter

*Soil Collection*: Simultaneous with plant sampling, rhizosphere soil samples were collected and allocated into three categories for distinct analyses: total nutrient content, available nutrients, and soil enzyme activities. Each soil sample was air-dried and sieved through a 100-mesh screen to remove organic debris prior to further analysis.

*Soil Enzyme Activities*: Urease (Ure) activity was determined by measuring the nitrate nitrogen content after incubation (Fei et al., 2020). Sucrase (SC) activity was quantified based on the amount of glucose released per gram of dry soil over a 24-hour period (Gessler et al., 2017).

*Elemental Nutrients:* Soil samples underwent wet digestion in a graphite furnace at 300°C using H_2_SO_4_ until fully digested. The mineral composition of these samples was analyzed using methodologies consistent with those described for plant tissues in Section 2.4, ensuring comparability in the assessment of nutrient status.

*Available Nutrients*: Nutrient extractions were performed using a 1 mol·L^-1^ KCl solution for ammonia nitrogen (NH^4+^-N, AN) and nitrate nitrogen (NO^3−^-N, NN). Available phosphorus (AP) was extracted using NaHCO_3_, and concentrations were determined by measuring absorbance at 700 nm (Fei et al., 2020).

### 2.6 Statistical analysis

Preliminary data analysis was performed using SPSS Statistics 26.0 (IBM). Differences among treatment groups were evaluated using Duncan’s Multiple Range Test to identify statistically significant variations. We utilized the General Linear Model (GLM) in SPSS to analyze the individual and interactive effects of nitrogen (N) and water (W) on the targeted response variables. In the Mantel test, pairwise comparisons of various factors are analyzed using Pearson’s correlation coefficient. For further data analysis and visualization, R software (Version 4.3.1) was employed. Principal Component Analysis (PCA) was conducted using the ‘prcomp’ function and visualized with the ‘ggbiplot’ package. Redundancy Analysis (RDA) and Mantel tests were executed using the ‘vegan’ package. The ‘ggplot2’ package facilitated the creation of histograms, radar charts, and the visualization of PCA, RDA results, and network diagrams illustrating the Mantel test outcomes.

## 3 Results

### 3.1 Plant growth

**Figure 1:**
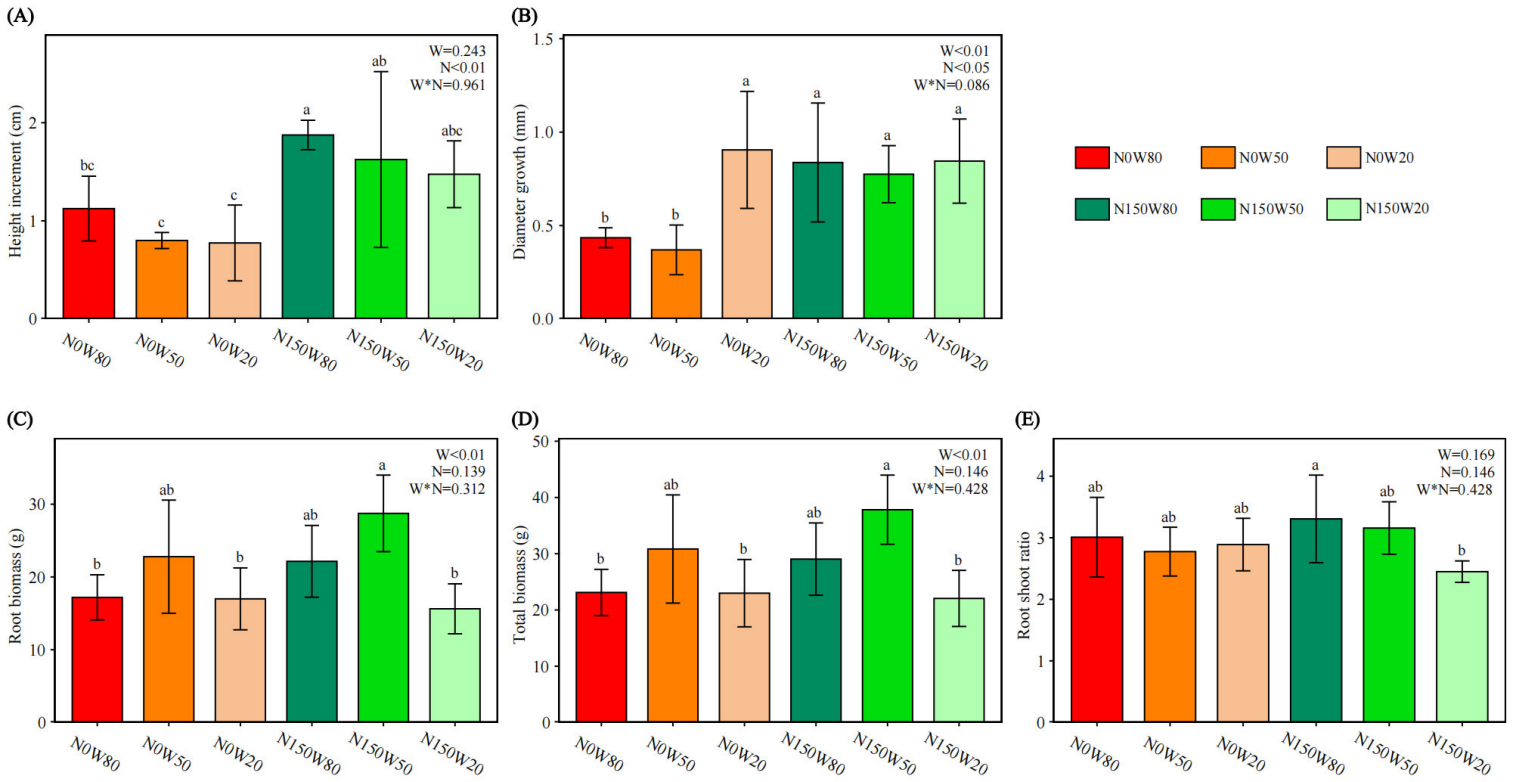
Effects of N and water treatments on the growth of *Quercus dentata*. **(A)** Height increment, **(B)** Stem diameter, **(C)** Root biomass, **(D)** Total biomass, and **(E)** Root shoot ratio. The horizontal axis represents the combinations of N (0 and 150kg·ha^−1^·yr^−1^) and W (80%, 50%, and 20% saturation soil moisture content). Error bars represent the standard deviation of the mean (n = 4). Statistically significant differences between treatments are indicated by lowercase letters above each bar, as determined by Duncan’s *post-hoc* test (*P* ≤ 0.05). General linear model was applied to examine the individual and interactive effects of N and water on different parameters.

The addition of N significantly enhanced the height of *Q. dentata* seedlings under W50 and W80 conditions (**Figure 1A**). In contrast, no significant growth in height was observed under W20 conditions with N treatment. Similarly, stem diameter growth was positively influenced by N application under W50 and W80, exhibiting a marked increase in diameter. However, under W20 conditions, notable stem growth was only evident in the absence of N application (**Figure 1B**).

The trends in aboveground and belowground biomass of trees are similar, with the highest biomass occurring in the N150W50 treatment (**Figures 1C, D**). This is significantly higher than all W20 treatments and also significantly exceeds the N0W80 treatment. There is a significant difference in the root shoot ratio between the highest ratio observed in N150W80 and the lower ratios in N150W20 (**Figure 1E**).

### 3.2 Gas exchange and chlorophyll content

**Figure 2:**
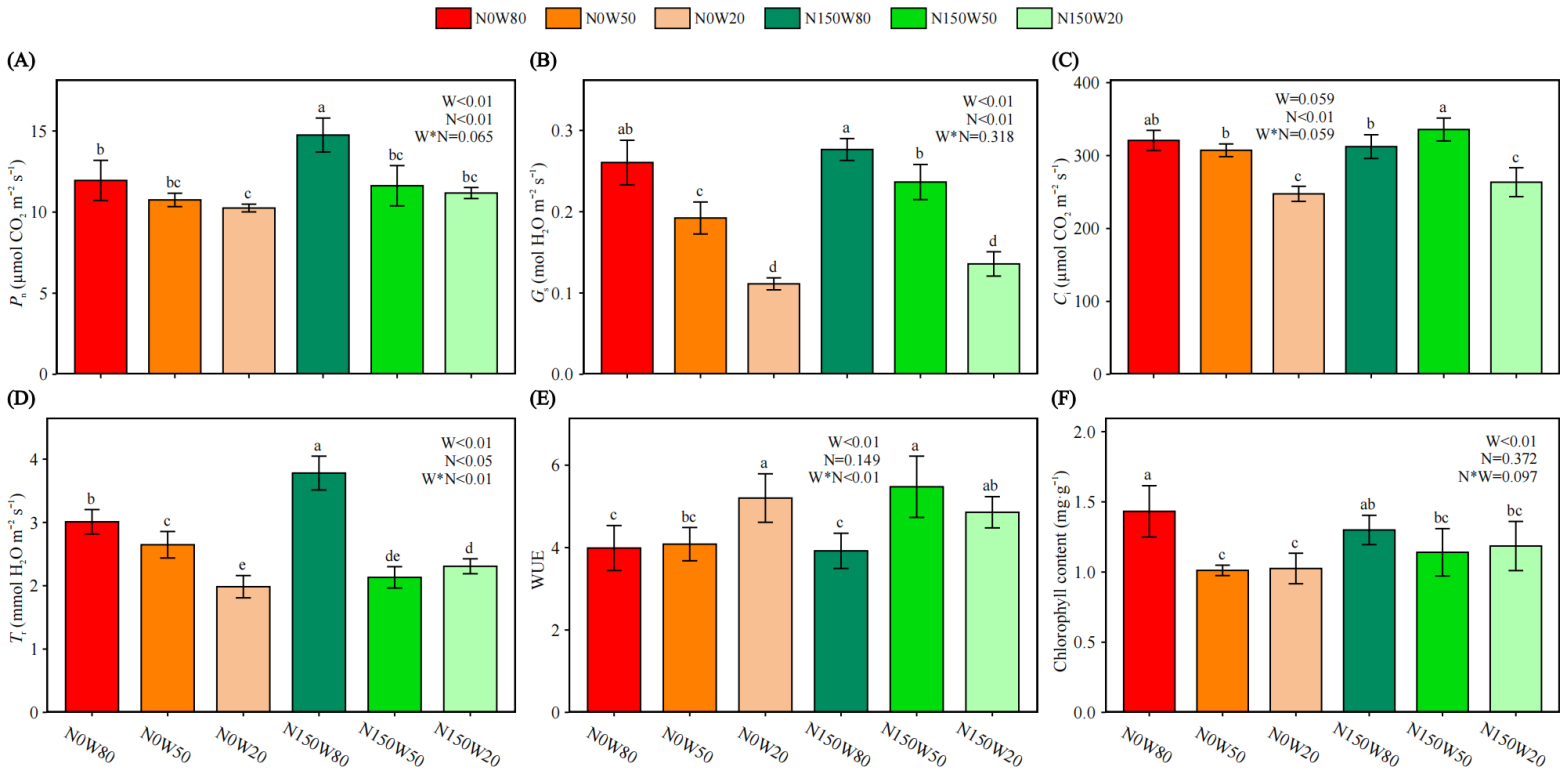
Effects of N and water treatments on the photosynthesis of *Quercus dentata*. **(A)** Net photosynthetic rate (*P*_n_), **(B)** Stomatal conductance (*G*_s_), **(C)** Intercellular CO_2_ concentration (*C*_i_), **(D)** Transpiration rate (*T*_r_), **(E)** Water use efficiency (WUE) and **(F)** Chlorophyll content. The different color represents the combinations of N (0 and 150kg·ha^−1^·yr^−1^) and W (80%, 50%, and 20% saturation soil moisture content). Error bars represent the standard deviation of the mean (n = 4). Statistically significant differences between different treatments at the same time are indicated by lowercase letters above each bar, and between the same treatments at different times are indicated by uppercase letters above each bar, as determined by Duncan’s *post-hoc* test (*P* ≤ 0.05). General linear model was applied to examine the individual and interactive effects of N and water on different parameters.

As relative water content (RWC) decreased, *P*_n_ similarly declined, with the highest values under N150 at the W80 moisture level (**Figure 2A**). *G*_s_ significantly decreasing with reduced RWC but remaining higher under N150 compared to N0 at equivalent moisture levels (**Figure 2B**). Although *C*_i_ reaches its peak under the N150W50 treatment, overall, *C*_i_ decreases as RWC declines, with a significant downward trend, especially under severe drought conditions (**Figure 2C**). *T*_r_ also shows a significant decline as RWC decreases, with the highest *T*_r_ observed in the N150W80 treatment. Except for the N150W50 treatment, where *T*_r_ is significantly lower than N0 at the same RWC, *T*_r_ is significantly higher under N150 treatments in other cases (**Figure 2D**). The addition of N150 results in the highest WUE under the W50 among all treatments (**Figure 2E**). However, regardless of the presence of additional N, severe drought consistently results in a significantly higher WUE compared to the W80 treatment(**Figure 2E**). Chlorophyll content, indicative of photosynthetic potential, significantly decreases under the N0 drought treatment, and there are no significant differences across all drought treatments (**Figure 2F**). The chlorophyll content under the N150 drought treatment was significantly lower than that of N0W80 (**Figure 2F**).

### 3.3 Cell membrane permeability and regulatory substances content

Leaf water content exhibited a slight decrease with reduced RWC under the N0 treatment. This reduction was notably more pronounced under the N150 treatment, particularly under W80 and W20 conditions, where N150 led to significantly higher water content compared to N0 (**Supplementary Figure S1A**). As indicators of cell membrane permeability and cellular stress response, REC levels showed significant variations influenced by both water availability and N treatment. Under N0, REC levels were highest under W20. Conversely, N150 generally maintained lower REC levels, with the lowest levels significantly noticeable at W50. Under W20, REC was significantly reduced with N150 compared to N0 (**Supplementary Figure S1B**). Malondialdehyde content was only significantly lower in both W50 treatments compared to N0W80 (**Supplementary Figure S1C**). N150 significantly reduced SP content under W20 but enhanced it under W50 and W80 levels compared to N0 treatment (**Supplementary Figure S1D**). SS content exhibited an increasing trend with decreased RWC under the N0 treatment, showing significant variations between W80 and W20 conditions (**Supplementary Figure S1E**). Proline content increased as RWC decreased. Under the N0 treatment, Pro content was significantly higher at W20 level compared to W80 condition. With the N150 treatment, Pro levels were significantly elevated across all moisture levels, showing a marked increase as RWC decreased, even when compared to the N0 treatment (**Supplementary Figure S1F**).

### 3.4 Antioxidant enzyme activity

Under the N0 condition, SOD activity was significantly higher under W80 and W20 compared to W50 (**Supplementary Figure S2A**). In contrast, under the N150 condition, the highest SOD activity was observed at W80, with notably higher activity at W20 than W50. Under the N0W20 treatment, POD were significantly higher than those in N150W50 (**Supplementary Figure S2B**). Under the N0 treatment, CAT activity was higher at W50 than in W80, with a slight, though non-significant, increase under W20 compared to W80 (**Figure S2C**). Under the N150 treatment, CAT activity was modestly elevated at W80 compared to W20 and was significantly higher than at W50. Across comparable water conditions, CAT activity under N150 consistently exceeded that observed under the N0 condition.

### 3.5 Soil enzyme activity and available nutrient content

**Figure 3:**
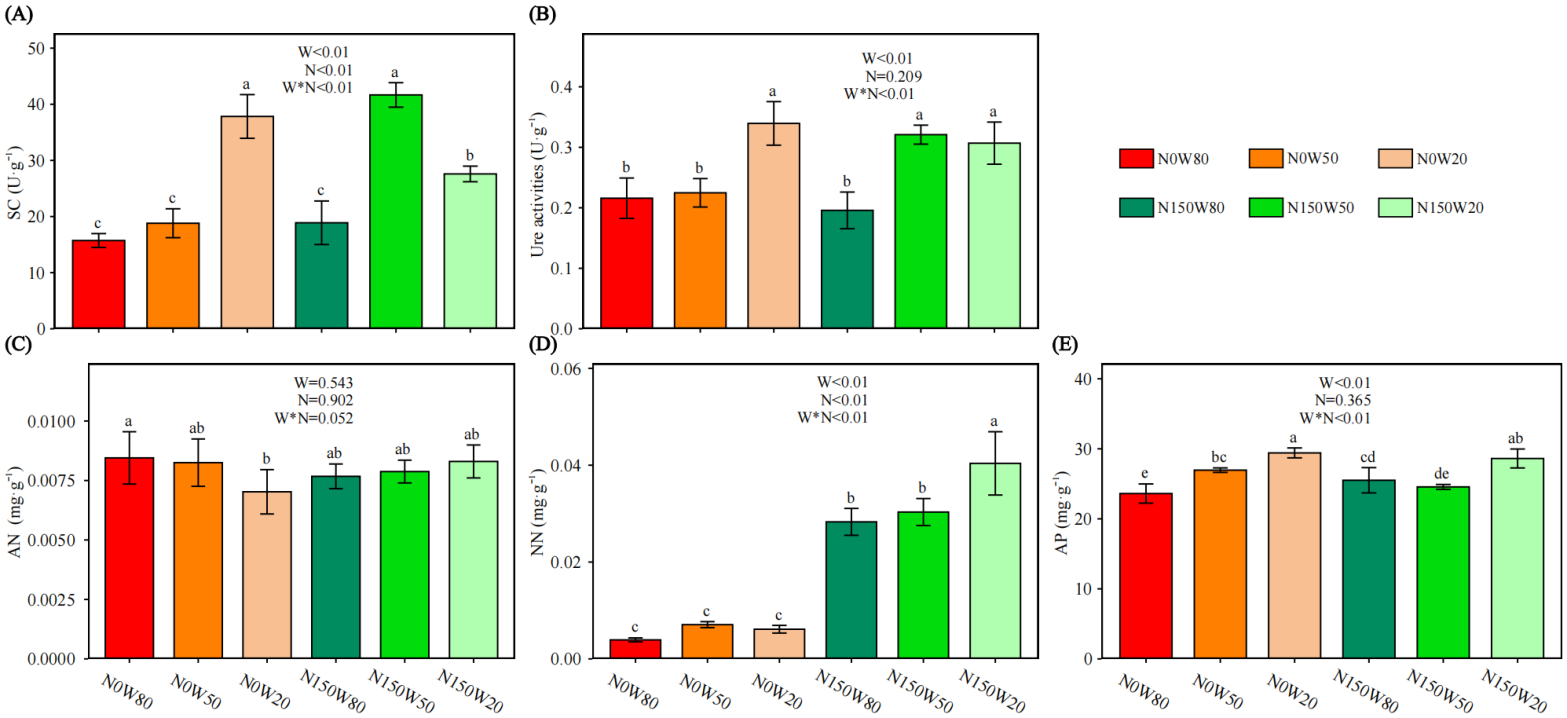
Effects of N and water treatments on the soil enzyme activity of *Quercus dentata*. **(A)** Sucrase (SC), **(B)** Urease (Ure) activity, **(C)** Ammonia nitrogen (AN), **(D)** Nitrate nitrogen (NN), and **(E)** Available phosphorus (AP). The horizontal axis represents the combinations of N (0 and 150kg·ha^−1^·yr^−1^) and W (80%, 50%, and 20% saturation soil moisture content). Error bars represent the standard deviation of the mean (n = 4). Statistically significant differences between treatments are indicated by lowercase letters above each bar, as determined by Duncan’s *post-hoc* test (*P* ≤ 0.05). General linear model was applied to examine the individual and interactive effects of N and water on different parameters.

Under the N0 treatment, SC activity increased as soil moisture decreased, with the highest activity observed under W20 (**Figure 3A**). Conversely, under the N150 treatment, SC activity was most pronounced at W50 and was significantly lower under W80 compared to W20. Notably, SC activity at W50 was higher under N150 than at the same moisture level under N0, but it significantly decreased under W20 with N150 compared to the N0 condition.

Without N supplementation, Ure activity remained stable at W50 but increased significantly under W20 compared to W80 (**Figure 3B**). With N150 treatment, Ure activity increased with decreasing RWC, peaking under W50. Additionally, at W50, Ure activity was significantly higher under N150 than under the N0 condition.

Under the N0 condition, AN levels decreased with diminishing soil moisture, with a significant reduction observed under W20 compared to W80 (**Figure 3C**).

Without N supplementation, NN content showed little variation across different soil moisture levels (**Figure 3D**). However, NN levels under N150 were significantly elevated in comparison to all other conditions, peaking under W20.

Under the N0 treatment, AP levels increased as RWC decreased, peaking under W20 (**Figure 3E**). Conversely, under the N150 treatment, AP at W20 was significantly higher than at other water stress levels. Comparatively, AP content under N150W80 was significantly greater than at N0W80, while under N150W50, it was significantly lower than under N0W50.

### 3.6 Plant and soil nutrient elements content

**Figure 4:**
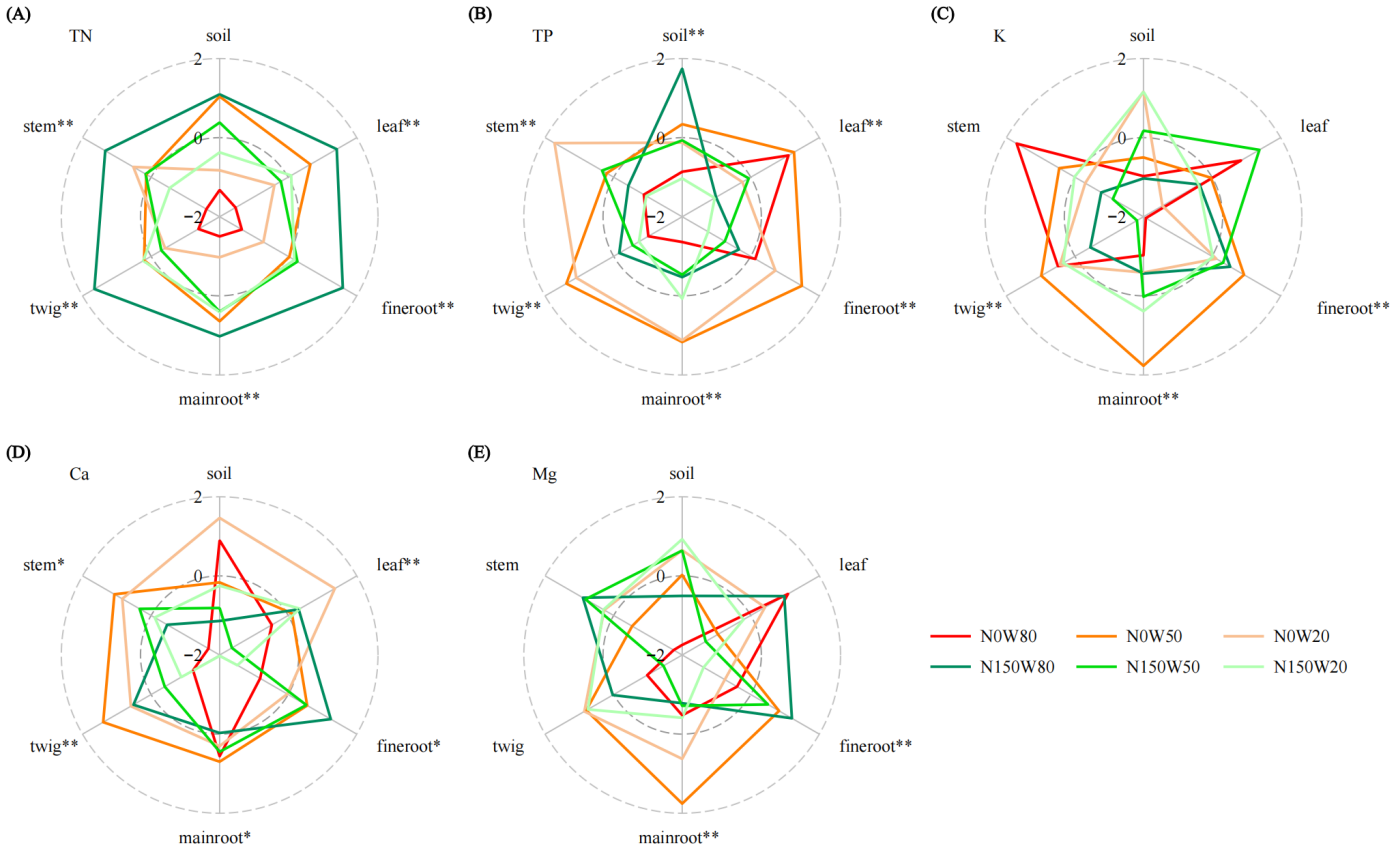
Effects of N and water treatments on the plant and soil nutrient elements of *Quercus dentata*. **(A)** Total nitrogen (TN), **(B)** Total phosphorus (TP), **(C)** Potassium (K), **(D)** Calcium (Ca), and **(E)** Magnesium (Mg) content. The different colors represents the combinations of N (0 and 150kg·ha^−1^·yr^−1^) and W (80%, 50%, and 20% saturation soil moisture content). General linear model was applied to examine the individual and interactive effects of N and water on different parameters (*P ≤ 0.05; **P ≤ 0.01).

Soil TN content under the N150W80 and N0W50 treatments was considerably higher compared to the N0W80 treatment (**Figure 4A**; **Supplementary Table S1**), and in plant tissues, TN concentrations were highest under the N150W80 treatment across all measured tissues, while the lowest TN content was recorded under the N0W80 treatment. Soil TP under the W80 condition was significantly greater with N150 application compared to N0. Contrarily, within plant tissues, TP content decreased under increased N loading, especially in leaves and fine roots under the W80 condition (**Figure 4B**; **Supplementary Table S1**). K content was highly responsive to water availability and N levels, particularly noted in roots and tender twigs (**Figure 4C**; **Supplementary Table S1**). The highest soil K content occurred under the W20 condition, decreasing as RWC increased. Despite this soil trend, K content in plant tissues generally increased with N application, except in the stems. The interaction between water and N significantly affected Ca content across various plant parts, with notable impacts observed in leaves and twigs under the W20 and W50 treatments without N loading, respectively (**Figure 4D**; **Supplementary Table S1**). There was a marked decrease in Ca content in leaves and main roots under the W50 and W20 treatments with N loading, respectively. Ca content in the soil remained relatively stable. Mg content was significantly influenced by the interplay of water and N, particularly in the roots of *Q. dentata* (**Figure 4E**; **Supplementary Table S1**). There was a notable decrease in Mg content in leaves under W20 conditions following N application. Conversely, fine roots and stems showed an increase in Mg content under conditions of ample water supply with the N150 treatment compared to the N0 condition. Soil Mg content exhibited minimal variation but was generally higher under the N150 treatment.

**Figure 5:**
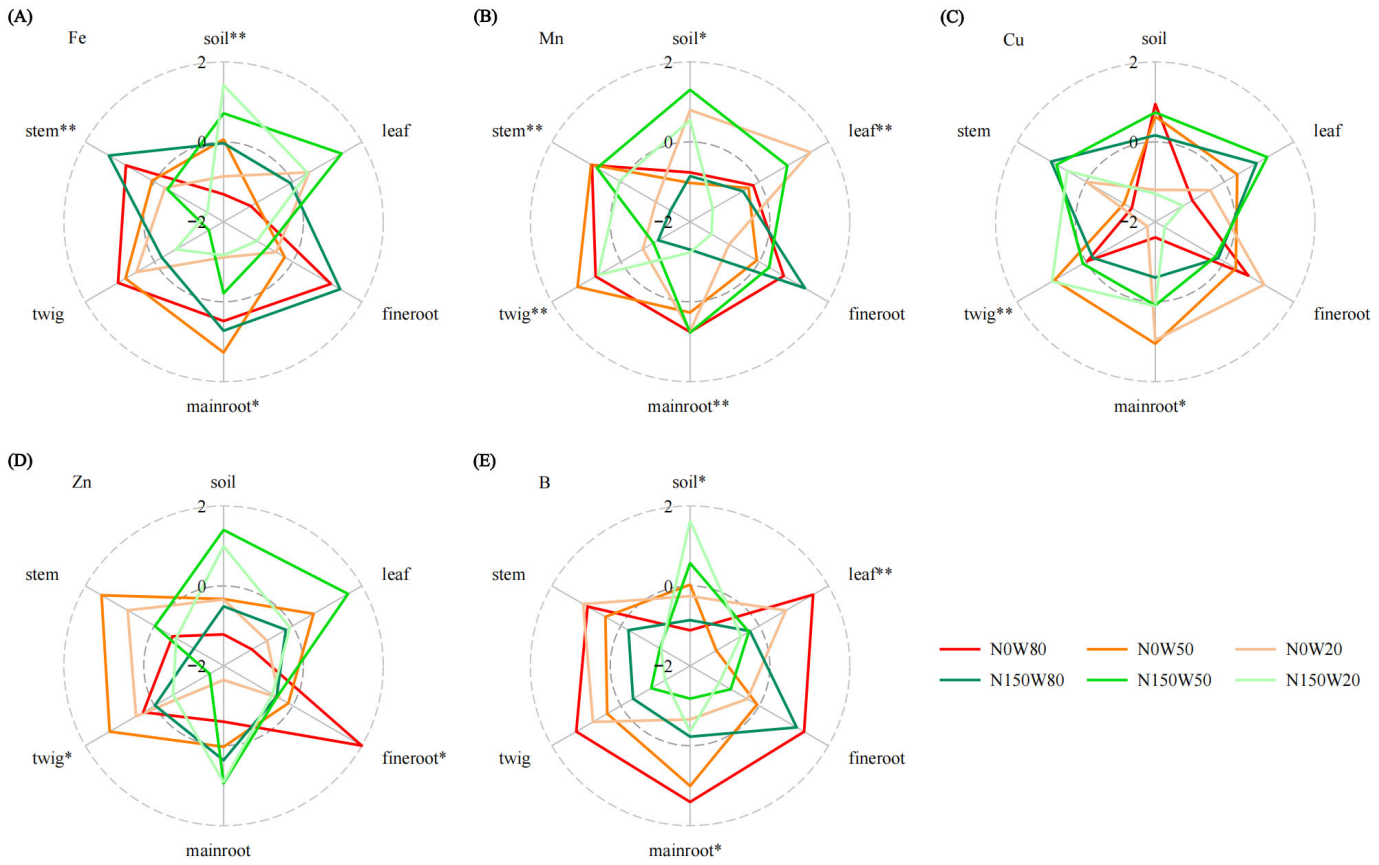
Effects of N and water treatments on the plant and soil nutrient elements of *Quercus dentata*. **(A)** Iron (Fe), **(B)** Manganese (Mn), **(C)** Copper (Cu), **(D)** Zinc (Zn), and **(E)** Boron (B) content. The different colors represents the combinations of N (0 and 150kg·ha^−1^·yr^−1^) and W (80%, 50%, and 20% saturation soil moisture content). General linear model was applied to examine the individual and interactive effects of N and water on different parameters (*P ≤ 0.05; **P ≤ 0.01).

The interaction between water availability and N levels significantly influences Fe content in both soil, main root and plant stems. Specifically, soil Fe content under N150W20 treatment is significantly higher than at N0 levels, while reductions are notable under both the N0W50 and N150W80 conditions (**Figure 5A**; **Supplementary Table S1**). Mn content in soil and various plant tissues, including leaves, main roots, twigs, and stems, are markedly affected by both water availability and N application (**Figure 5B**, **Supplementary Table S1**). Soil Mn content is significantly enhanced under the moderate water stress with N150W50. Meanwhile, Mn concentrations in branches are significantly higher under the N150W20 treatment compared to the N0W20, though this trend reverses in other plant tissues (**Figure 5B**; **Supplementary Table S1**). Cu content shows distinct variations in main roots and twigs, particularly under the N150 treatment, which appears to alter Cu accumulation in response to different water levels (**Figure 5C**; **Supplementary Table S1**). While soil Cu content is higher under the N0W80 compared to N150W20, changes in RWC or N150 treatment do not significantly alter overall soil Cu levels (**Figure 5C**; **Supplementary Table S1**). Zn content in fine roots and twigs are substantially influenced by water and N conditions, with a significant increase in Zn content observed in main roots under N150W20 treatment (**Figure 5D**; **Supplementary Table S1**. Fine root Zn content is considerably elevated under the N0W80 treatment compared to other treatments. Conversely, soil Zn content is significantly lower under the N0W80 treatment compared to high N treatments at W50 and W20 conditions (**Figure 5D**; **Supplementary Table S1**). B content in soil, main roots, and leaves is profoundly impacted by both water availability and N (**Figure 5E**; **Supplementary Table S1**). As RWC decreases, soil B content increases, particularly under the N150W20 treatment, while a notable decrease is observed under the N0W80. In plant tissues, the N150 generally reduces B content across all water conditions (**Figure 5E**; **Supplementary Table S1**).

### 3.7 Relationships of plant growth and soil properties

**Figure 6:**
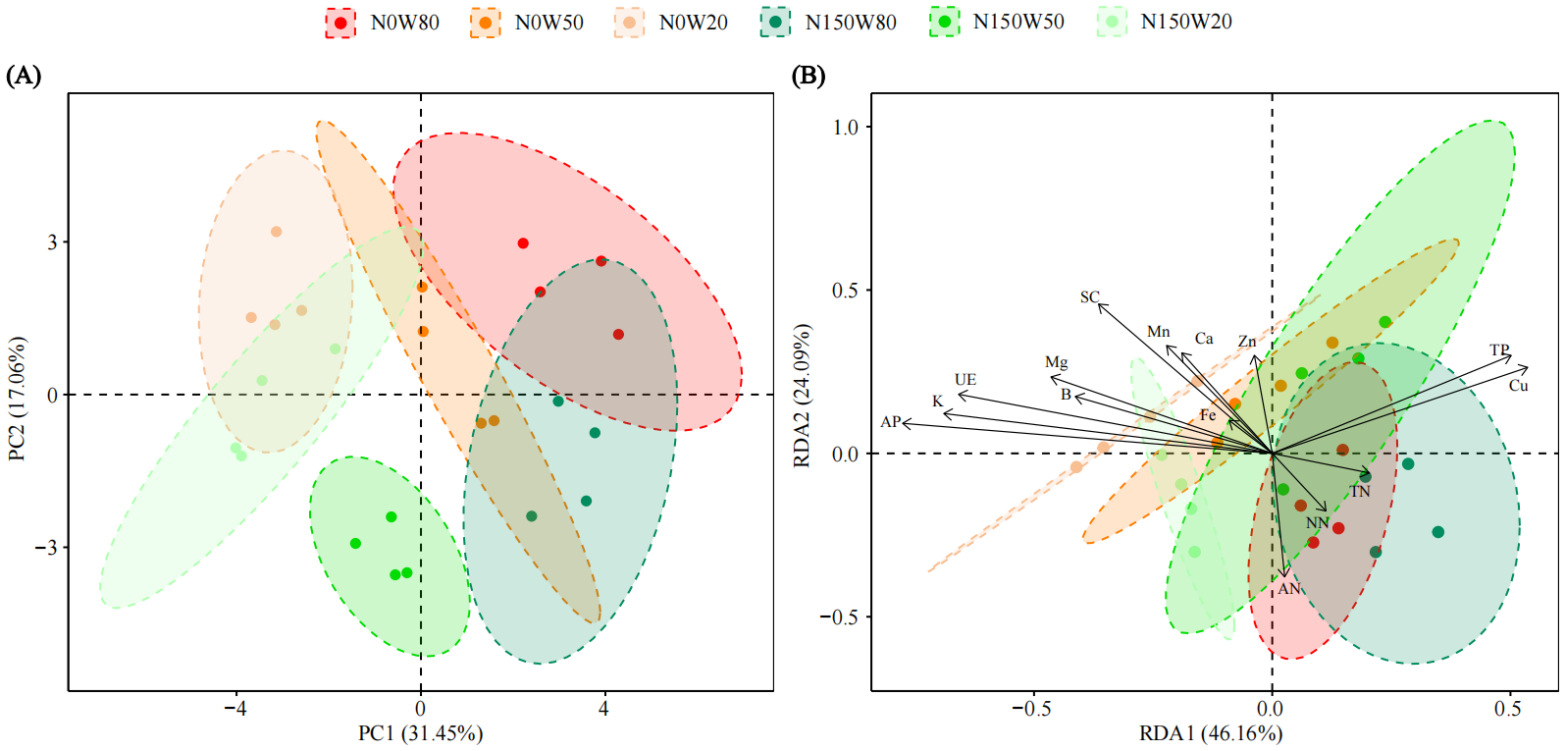
Principal Component Analysis (PCA, **A)** illustrating the effects of N and water treatments on the growth and soil parameters of *Quercus dentata*. Distribution pattern of plant growth indices and physicochemical parameters by redundancy analysis (RDA, **B)**. Plant growth indices include Height increment, Stem diameter, Root shoot ratio, Root biomass, Total biomass, Chlorophyll content, Net photosynthetic rate (*P*_n_), Stomatal conductance (*G*_s_), Intercellular CO_2_ concentration (*C*_i_), and Transpiration rate (*T*_r_). Soil physicochemical properties include Ammonia nitrogen (AN), Nitrate nitrogen (NN), Available phosphorus (AP), Total nitrogen (TN), Total phosphorus (TP), Potassium (K), Calcium (Ca), Magnesium (Mg), Iron (Fe), Manganese (Mn), Copper (Cu), Zinc (Zn), Boron (B), Urease (Ure), and Sucrase (SC). The different colors represents the combinations of N (0 and 150kg·ha^−1^·yr^−1^) and W (80%, 50%, and 20% saturation soil moisture content).

The PCA revealed that the first principal component (PC1) accounted for 31.45% of the total variance, while the second principal component (PC2) explained an additional 17.06%. The parameters were distinctively grouped and arrayed across the PCA plot (**Figure 6A**). The treatment group subjected to severe drought was positioned negatively along PC1. In contrast, the group with medium soil moisture concentration was centrally located, and the group with optimal soil moisture appeared positively on PC1 (**Figure 6A**).

This PCA outcome underscores that drought conditions substantially affect plant physiological traits, with N supplementation modifying these effects, although not uniformly across different levels of water availability (**Figure 6A**). Significant variables influencing PC1 included Ure and K, which exhibited strong positive loadings. In contrast, *G*_s_ and *T*_r_ showed pronounced negative loadings (**Supplementary Tables S2**, **S3**). For PC2, the primary positive contributors were total biomass, root biomass, NN, and Zn, each with substantial positive loadings (**Supplementary Tables S2**, **S3**).

Redundancy analysis was employed to explore the intricate interactions between the growth indicators of *Q. dentata* and various soil physicochemical factors. The analysis, as detailed in **Figure 6B**, indicates that the first redundancy axis (RDA1) explains 46.16% of the variance, while the second axis (RDA2) accounts for an additional 24.09%. The clustering of treatment groups on the plot delineates the distribution of treatment effects across different soil moisture and N levels.

In the biplot, treatment groups with higher water availability, such as N0W80 and N150W80, are positioned towards the positive direction of RDA1. Conversely, groups under severe drought conditions, such as N0W20 and N150W20, are positioned towards the negative direction of RDA1, reflecting the stress impacts on plant physiology.

Key elements like Cu and TP are positioned towards the positive end of RDA1, suggesting their higher concentrations are associated with specific treatment conditions (N0W80, N150W80 and N150W50). AP (*F*=2.638, *P*=0.046) along with AN, shows vectors pointing towards the negative direction of RDA1, indicating a decrease or stress-associated reduction in these nutrients under certain conditions (N0W50, N0W20, N150W50 and N150W20).

Important micronutrients such as Fe and Ca align closely with the RDA1 axis, highlighting their variation across different water and N conditions, which may reflect their critical roles in plant stress response and nutrient uptake. The plot also includes vectors for Mg and SC activity, which are less aligned with the primary gradient. This suggests that their influences are modulated by a combination of factors not solely explained by N or water levels.

### 3.8 Relationship between soil physicochemical properties and the plant nutrient uptake

**Figure 7:**
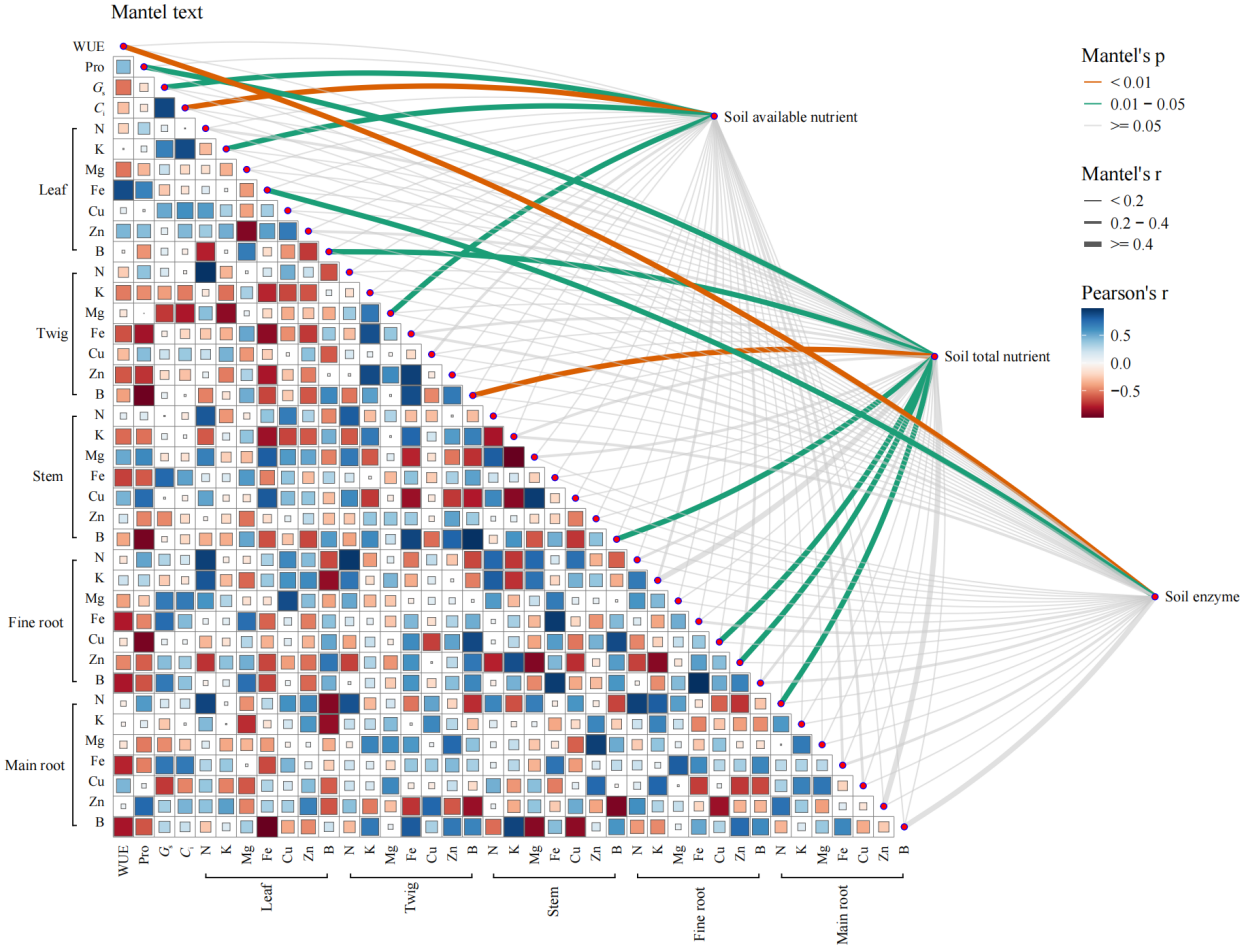
Analysis of the correlations soil physicochemical properties and the plant physiology and nutrient under N and water treatments. Pairwise comparisons of the analyzed factors are displayed with a color gradient denoting Pearson correlation coefficients. Available nutrient, total nutrient and enzyme in the rhizosphere soil were related to each variable by Mantel test. The plant physiology and nutrient include Water use efficiency (WUE), Chlorophyll content, Leaf water content (WC), Relative electrical conductivity (EC), Malondialdehyde (MDA) content, Soluble protein (SP) content, Soluble sugar (SS) content, Proline (Pro) content, Superoxide dismutase (SOD), Peroxidase (POD), Catalase (CAT) activity, Net photosynthetic rate (*P*_n_), Stomatal conductance (*G*_s_), Intercellular CO_2_ concentration (*C*_i_), and Transpiration rate (*T*_r_), Total nitrogen (TN), Total phosphorus (TP), Potassium (K), Calcium (Ca), Magnesium (Mg), Iron (Fe), Manganese (Mn), Copper (Cu), Zinc (Zn), Boron (B). Soil available nutrient include Ammonia nitrogen (AN), Nitrate nitrogen (NN), available phosphorus (AP). Soil total nutrient include TN, TP, K, Ca, Mg, Fe, Mn, Cu, Zn, (B) And soil enzyme include Urease (Ure), and Sucrase (SC).

Mantel test identifies specific relationships with soil-available nutrients. Notably, variables such as *G*_s_, *C*_i_, leaf K, and twig Mg are all directly influenced by the availability of nutrients in the soil (**Figure 7**). In contrast, a broader spectrum of variables including Pro, B in leaves and twigs, Cu in fine roots, Zn in fine roots, and N in main roots, correlates strongly with the total nutrient content of the soil. Soil enzyme activity has a limited impact on *Q. dentata*, showing significant correlations only with WUE and leaf Fe content. It is evident that the impact of changes in soil physicochemical properties on *Q. dentata* is more pronounced in the leaf component (**Figure 7**). Furthermore, the heatmap analysis reveals that plant physiological traits and macroelements, such as Leaf water content, REC, MDA, SP, SS, SOD, POD, CAT, *P*_n_, *T*_r_, Chlorophyll content, P, Ca, and Mn, do not exhibit significant correlations with the measured soil physicochemical indicators and are thus omitted here.

## 4 Discussion

### 4.1 Effects of nitrogen deposition and drought stress on *Q. dentata* growth

Our findings highlight that N150 significantly boosts plant height, stem diameter, and overall biomass, particularly noticeable under W50. This enhancement is not mirrored under W20, indicating that N’s effectiveness is contingent upon adequate hydration (**Figures 1A–C**). While N drives morphological changes such as the root shoot ratio, it does not solely govern biomass accumulation, which is primarily influenced by water availability (Li et al., 2023b). This suggests that N’s role in morphogenesis, although significant, culminates in productivity only under sufficient water conditions. We found that the interaction between N and water availability subtly influences the growth dynamics of *Q. dentata*. Biomass enhancements were observed at W50 and W80 when combined with N150 treatment. This implies that N potentially optimizes WUE, facilitating improved physiological adaptations that bolster plant productivity under varied hydration levels (Plett et al., 2020; Wang et al., 2024). Contrastingly, severe water stress limits the beneficial impacts of N, underscoring the pivotal role of water in growth dynamics, regardless of N levels.

Nitrogen supplementation was found to alleviate the adverse effects of moderate drought on photosynthesis, as evidenced by the reduced inhibition of *G*_s_ (**Figure 2B**). The Mantel test further revealed a positive correlation between soil available nutrient with the regulation of stomatal behaviors, enhancing gas exchange under stress conditions (**Figures 2B, C**, **3D, E**, **7**). These interactions suggest complex physiological adjustments that facilitate survival and productivity under environmental stresses (Gessler et al., 2017). Furthermore, the stability of chlorophyll content under N150 across different watering regimes suggests N’s protective role against degradation in stressful conditions (Wang et al., 2024). This stability is crucial for maintaining photosynthetic efficiency, which is significantly enhanced in well-watered conditions under N150, leading to superior growth performance (**Figure 2F**). Unexpectedly, soil enzyme activity exhibited a striking similarity to changes in WUE, particularly under the N150 conditions (**Figures 2E**, **3A, B**, **7**). This finding indicates that N not only enhances drought resistance but also boosts WUE, aligning with improved plant productivity. However, the absence of biomass increase under W20 despite N application suggests that the threshold of water stress may negate N’s benefits, highlighting the energy-intensive nature of maintaining physiological functions under extreme conditions (Liang et al., 2020).

During the growing season, initial increases in Tr and subsequent declines in WUE under normal water conditions without N supplementation indicate that while roots may efficiently absorb water, inadequate N may restrict photosynthetic capacity, leading to inefficient water use (Farshad et al., 2023). As the season progresses, plants adjust their physiological strategies, potentially over-relying on transpiration to regulate internal processes under high N conditions, which might not always correlate with efficient growth and biomass accumulation. This suggests a complex balance between water and N management to optimize photosynthetic activity and plant productivity (Luo et al., 2023). Meanwhile, the dynamics of *C*_i_ and its relationship with *G*_s_ underscore the intricate mechanisms regulating CO_2_ uptake and water loss. Under optimal conditions, enhanced N availability likely improves CO_2_ utilization within the photosynthetic machinery, optimizing the balance between CO_2_ influx and photosynthetic demands (Xiong et al., 2015; Liu et al., 2021; Zhang et al., 2021a).

### 4.2 Effects of nitrogen deposition and drought stress on physiology of *Q. dentata*

Our study delineated significant interactions between N deposition and water availability, profoundly affecting the physiological and biochemical parameters of *Q. dentata*. Notably, leaf water content was responsive to these environmental variables, decreasing with diminished soil moisture yet increasing significantly under N150 treatments at both W80 and W20 water stress levels (**Supplementary Figure S1A**). This enhancement underlines the synergistic impact of N in conjunction with sufficient water in sustaining cellular hydration and optimizing WUE. Meanwhile, as the proxy for cellular ion leakage, EC was elevated under N0W20 compared to N0W80, suggesting that lower water availability exacerbates membrane permeability (**Supplementary Figure S2B**). However, under N150, this trend was abated, indicating enhanced membrane integrity and reduced ion leakage, potentially due to N-mediated cellular stabilization (Liang et al., 2022).

This physiological process can also be explained by changes of lipid peroxidationin and antioxidant enzyme. In our study, MDA levels remained relatively stable under N150 across varying water regimes. This stability is pivotal in preserving cellular function against the typical increase in membrane permeability under drought stress, thus mitigating cellular damage (Peguero-Pina et al., 2014). However, the activity of SOD, a critical antioxidant enzyme, displayed a biphasic response; it decreased initially under mild drought but increased under severe conditions (**Supplementary Figure S2A**). This suggests that while mild drought may not sufficiently trigger antioxidant defenses, acute stress conditions elicit a strong antioxidative response, enhanced by N supplementation (Hussain et al., 2019).

As for osmoregulatory substances, Pro significantly increased under N150 as soil moisture declined, illustrating N’s role in bolstering osmotic adjustment and metabolic support under stress (**Supplementary Figure S1F**). Conversely, the levels of SP and SS, which are integral to photosynthetic efficiency and energy storage, respectively, showed less variability. Higher SP levels was found under optimal N and water conditions, which may reflect enhanced synthesis of Rubisco, a critical enzyme for photosynthesis, thereby stabilizing photosynthetic capacity in our case (Evans, 1989; Makino et al., 2003). SS levels mirrored this trend, suggesting that the biochemical capacity for photosynthesis and energy storage was more constrained by water availability than by N enhancement (**Supplementary Figures S1D, E**). Interestingly, despite the physiological benefits conferred by N, our results suggest that under drought conditions, the energy derived from enhanced *P*_n_ may be diverted towards combating oxidative stress rather than growth, as indicated by the negative correlation between biomass accumulation and antioxidant enzyme activity (**Figure 7**) (Ullah et al., 2023).

Collectively, these findings highlight the complex interplay between N deposition and water availability in regulating the physiological processes of *Q. dentata*. The capacity of N to modulate EC, boost Pro synthesis, and influence SP levels under various water conditions underscores its vital role in enhancing plant stress tolerance and WUE, particularly in the context of changing environmental conditions.

### 4.3 Effects of nitrogen deposition and drought stress on physicochemical properties and nutrient uptake of *Q. dentata*

Nitrogen deposition and drought stress significantly affect soil enzyme activities and nutrient cycling, essential for ecosystem functionality and plant health, though these factors may not alter the community composition and activity of soil microorganisms (Kuypers et al., 2018; Rillig et al., 2023). Mantel test indicates a significant correlation between soil enzyme activity and WUE as well as leaf Fe content. This suggests that rhizosphere microorganisms and endophytic fungi significantly impact *Q. dentata* under environmental stresses, enhancing WUE and facilitating Fe transport, respectively. These microorganisms, however, also consume the plant’s photosynthetic products, potentially explaining the observed limitations in biomass accumulation. Furthermore, drought conditions constrain biochemical reactions and substance exchanges in the soil, which increases microbial enzyme activities to meet the heightened energy demands of both microbes and plants. This adaptive response likely accounts for the observed increases NN, AP, and TP of soil under drought conditions (**Figures 3D, E**, **4B**). Furthermore, the availability of N enhances enzyme production, thereby promoting organic matter decomposition and boosting nutrient availability, especially under restricted water conditions (Nouraei et al., 2022; Bender et al., 2023).

Additionally, changes in soil-available nutrients and *P*_n_ elucidate how plants resist environmental stress by energizing the soil microbial community, which in turn enhances nutrient availability through the secretion of extracellular enzymes. Urease activity, for instance, illustrates the interplay between N and water availability, intensifying under dry conditions while AN content decreases (**Figures 3B–D**). This phenomenon likely results from increased plant and microbial uptake and utilization under environmental stress or a rapid conversion of AN to other N forms (Gao et al., 2019). Enhanced microbial activity, a compensatory response to water stress, may also contribute to this dynamic, helping to maintain microbial metabolic functions (Dong et al., 2021). Conversely, under wet conditions, reduced Ure activity reflects more efficient N utilization by soil microbes (Zhang et al., 2023). The observed stability of AN content alongside increased NN content under N150 treatment suggests rapid absorption and nitrification, minimizing AN accumulation. Limitations in AP under N deposition, potentially due to enzymatic activity shifts within the P cycle, further complicate nutrient dynamics under drought (Cui et al., 2021; Wang et al., 2022). In our founding, a drought-induced reduction in WUE precipitates a decline in *T*_r_, which compromises the absorptive capacity of the root system and hinders the upward transport of nutrients from soil to plant. Additionally, soil water deficits restrict nutrient mineralization, thereby exacerbating the challenges associated with nutrient uptake by seedling roots. These observations suggest that under prolonged, though not extreme, water stress conditions, the limitation of nutrient supply may pose a greater constraint than water scarcity itself (Refsland and Fraterrigo, 2018).

As for nutrient uptake, plant physiological responses to these altered soil conditions demonstrate significant shifts in nutrient uptake and metabolism. In our study, enhanced TN content in various plant tissues typically bolsters growth and resilience under drought by improving WUE and nutrient uptake (Zhao et al., 2021; Qiu et al., 2022; Wei et al., 2022). This enhancement is especially pronounced under conditions combining optimal moisture with N enrichment, promoting efficient nutrient uptake and internal translocation. The interaction between N deposition and drought also impacts the absorption and distribution of macronutrients, with reduced K and Ca in leaves under drought, which negatively affects water retention and stomatal function. However, N does not significantly enhance Ca and Mg levels, indicating that these essential nutrients might be consumed during stress responses or photosynthetic processes rather than accumulating (Bose et al., 2011; Hochmal et al., 2015; Tatagiba et al., 2016). Trace elements respond variably to environmental conditions. Our findings indicate that drought impedes the uptake of Fe, Mn, Cu, and Zn, while nitrogen enhances their translocation, maintaining elevated levels in the leaves. Despite a reduction in Zn absorption, *Q. dentata* seedlings accumulate greater amounts of Zn in their enlarged taproots. This increase under drought conditions is likely facilitated by nitrogen fertilization, which not only promotes root expansion and branching but also enhances the roots’ capacity to absorb Zn. Concurrently, nitrogen and Zn exhibit a synergistic relationship, enhancing the plant’s demand for and efficiency in absorbing Zn (Cakmak, 2000; Li et al., 2013; Al-flahi, 2014; Wang et al., 2017; Li et al., 2021; Rijk et al., 2023). Moreover, Mantel test results reveal a significant correlation between the transported B in the aerial parts and the total B content in the soil (**Figures 5E**, **7**; **Supplementary Table S1**). Nevertheless, our research also demonstrates that the combined stresses of drought and nitrogen deposition, particularly during periods of drought, reduce the absorption and transport of B, suggesting that the prevailing environmental conditions may adversely affect the reproductive growth of *Q. dentata*.

Our study utilized PCA and RDA to distinguish the effects of water and N treatments on plant physiological, biochemical, and soil nutrient parameters. The clear separation between treatments highlights the significant impact of these factors on plant performance (**Figure 6A**). PCA analysis revealed that Ure and K have high positive loadings on the growth of *Q. dentata* (**Figure 6A**). RDA indicated that Cu, P, TN, and NN are the primary soil factors promoting the growth of *Q. dentata* (**Figure 6B**). The significant associations between soil nutrients and a variety of physiological and nutritional variables underscore that the effects of soil nutrient availability on plant health and development are broad and profound (Liu et al., 2024). Drought and soil eutrophication significantly changed the contents of various nutrients in the tissues, which highlighted the importance of balancing soil fertility for healthy growth of *Q. dentata*. However, compared to soil nutrients, the impact of soil enzymes on plant physiology may be indirect and more complex (Wołejko et al., 2020). Further research is necessary to elucidate the mechanisms through which soil enzymes influence plant health and to determine whether their influence varies under different environmental conditions.

## 5 Conclusion

This study delineates the intricate interplay between N deposition and drought stress on *Quercus dentata*’s growth and physiological adaptations. Enhanced N levels were particularly effective under N150W50, significantly boosting stem elongation, diameter, and overall biomass through improved *P*_n_, *G*_s_, and WUE. Compared to the N0W80 treatment, N150W50 significantly increased biomass, in stark contrast to the negligible effects observed with W20. This underscores the critical role of water as a primary driver of morphological changes. Moreover, N’s role in sustaining chlorophyll content and cellular integrity under drought supports persistent photosynthetic activity, particularly evident in the N150W80 treatment, where N and sufficient water synergistically enhance growth parameters and biomass accumulation. Furthermore, physiological analyses reveal N’s mitigation of drought’s adverse effects through increased leaf water content, stabilized cellular functions as evidenced by consistent electrical conductivity, and regulated antioxidant responses, particularly SOD. Additionally, N’s enhancement of Pro and SP supports better osmotic adjustment and nutrient assimilation, facilitating an improved physiological response to drought.

The interaction between N deposition and drought also profoundly impacts soil enzyme activities and nutrient cycling, with increased N availability boosting enzyme activity and nutrient availability under water stress. PCA and RDA further revealed the significant contributions of soil physicochemical properties, such as Ure, Cu, and N, to the growth of *Q. dentata*. This highlights the response mechanisms of *Q. dentata* to environmental changes. While N enhances nutrient uptake, particularly under optimal moisture conditions, drought conditions limit the effectiveness of this uptake, necessitating integrated management strategies to harness the benefits of N fully.

In conclusion, while N deposition can significantly bolster *Q. dentata*’s growth and physiological robustness under moderate drought, its efficacy is closely tied to water availability, highlighting the necessity for combined nutrient-water management to maximize plant productivity and ecological resilience. Further investigations are essential to unravel the long-term effects of these interactions on ecosystem health and plant physiological functionality.

## Data Availability

The raw data supporting the conclusions of this article will be made available by the authors, without undue reservation.
