# Supplementary material for: Adaptive growth strategies of Quercus dentata to drought and nitrogen enrichment: a physiological and biochemical perspective
Source: Front Plant Sci. 2024 Nov 22;15:1479563. doi: 10.3389/fpls.2024.1479563 (PMC11620892; doi:10.3389/fpls.2024.1479563)
Supplement: Supplementary file 1 [file DataSheet1.docx]

Supplementary Material

# Supplementary Figures and Tables

## Supplementary Figures

**
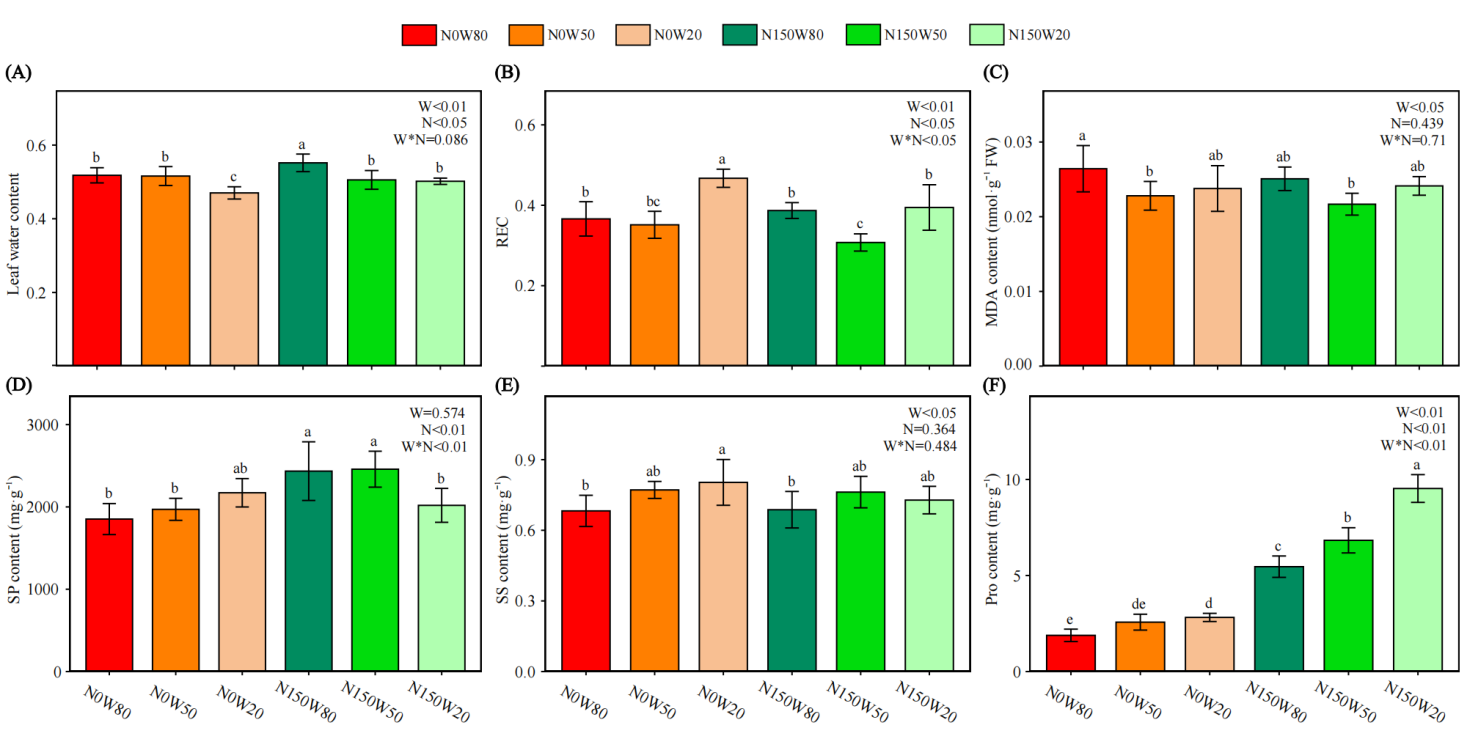
**

**Fig. S1** Effects of N and water treatments on the cell membrane permeability and regulatory substances of *Quercus dentata*. **(A)** Leaf water content, **(B)** Relative electrical conductivity (REC), **(C)** Malondialdehyde (MDA) content, **(D)** Soluble protein (SP) content, **(E)** Soluble sugar (SS) content, and **(F)** Proline (Pro) content. The horizontal axis represents the combinations of N (0 and 150kg·ha^−1^·yr^−1^) and W (80%, 50%, and 20% saturation soil moisture content). Error bars represent the standard deviation of the mean (n = 4). Statistically significant differences between treatments are indicated by lowercase letters above each bar, as determined by Duncan's post-hoc test (*P* ≤ 0.05). General liner model was applied to examine the individual and interactive effects of N and water on different parameters.

**
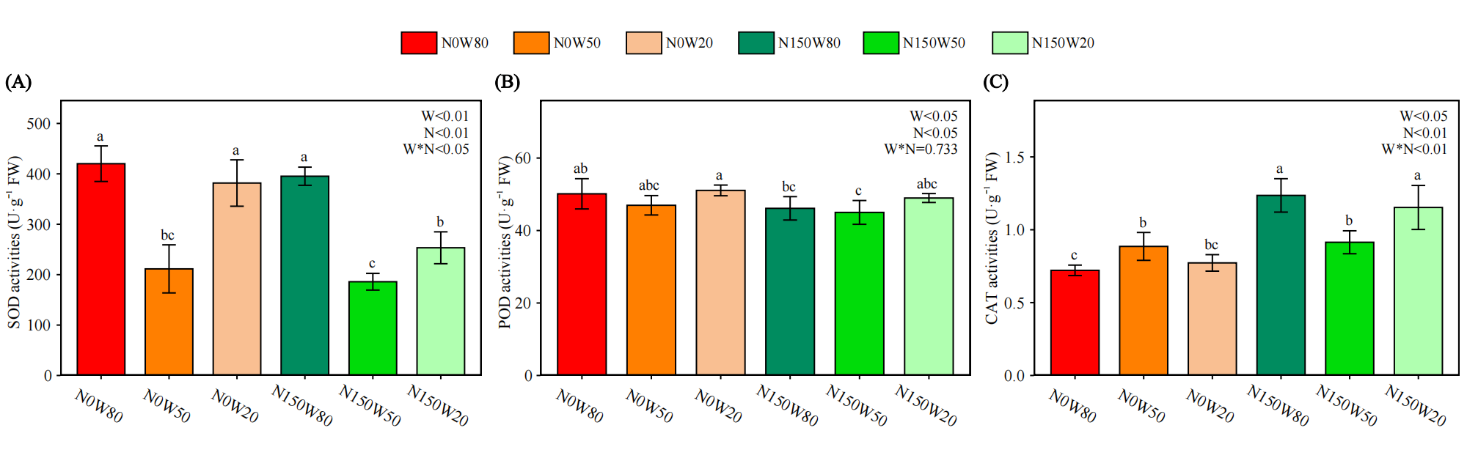
**

**Fig.S2** Effects of N and water treatments on the leaf antioxidant enzyme activity of *Quercus dentata*. **(A)** Superoxide dismutase (SOD), **(B)** Peroxidase (POD), and **(C)** Catalase (CAT) activity. The horizontal axis represents the combinations of N (0 and 150kg·ha^−1^·yr^−1^) and W (80%, 50%, and 20% saturation soil moisture content). Error bars represent the standard deviation of the mean (n = 4). Statistically significant differences between treatments are indicated by lowercase letters above each bar, as determined by Duncan's post-hoc test (*P* ≤ 0.05). General liner model was applied to examine the individual and interactive effects of N and water on different parameters.

## Supplementary Tables

**Table S1** Effects of N and water treatments on the plant and soil nutrient elements

| Index |  | Component | | | | |  |
| --- | --- | --- | --- | --- | --- | --- | --- |
|  | treatment | soil | leaf | fine root | main root | twig | stem |
| N(mg/g) | N0W80 | 1.455±0.057b | 17.513±0.527d | 10.166±0.344d | 4.965±1.336b | 7.04±0.638c | 6.552±0.302d |
|  | N0W50 | 1.709±0.033a | 22.138±1.192ab | 14.098±0.735b | 8.083±0.467a | 8.375±0.215b | 7.397±0.187b |
|  | N0W20 | 1.508±0.114ab | 19.914±1.177c | 11.952±1.21c | 5.733±0.607b | 7.86±0.598b | 7.579±0.17b |
|  | N150W80 | 1.715±0.21a | 23.769±0.577a | 18.521±0.585a | 8.637±1.099a | 9.597±0.52a | 7.974±0.278a |
|  | N150W50 | 1.638±0.2ab | 20.315±0.928c | 14.767±0.69b | 7.73±0.387a | 7.952±0.425b | 7.407±0.128b |
|  | N150W20 | 1.557±0.088ab | 20.954±1.891bc | 14.515±0.586b | 7.748±0.969a | 8.397±0.394b | 7.068±0.177c |
| *P* | W | 0.135 | 0.376 | 0.007 | 0.027 | 0.712 | 0.452 |
|  | N | 0.166 | 0.001 | 0 | 0 | 0 | 0.003 |
|  | W*N | 0.07 | 0 | 0 | 0.001 | 0 | 0 |
| P(mg/g) | N0W80 | 0.614±0.027b | 2.146±0.142a | 1.468±0.028c | 0.534±0.035c | 0.708±0.028b | 0.593±0.064c |
|  | N0W50 | 0.672±0.06ab | 2.208±0.165a | 1.81±0.056a | 0.936±0.068a | 0.989±0.078a | 0.706±0.105b |
|  | N0W20 | 0.649±0.063b | 1.631±0.129b | 1.615±0.216b | 0.929±0.069a | 0.955±0.095a | 0.866±0.035a |
|  | N150W80 | 0.74±0.028a | 1.336±0.027c | 1.347±0.012cd | 0.675±0.11b | 0.808±0.08b | 0.64±0.027bc |
|  | N150W50 | 0.652±0.055b | 1.702±0.098b | 1.244±0.016de | 0.665±0.052b | 0.762±0.095b | 0.72±0.103b |
|  | N150W20 | 0.606±0.037b | 1.321±0.035c | 1.121±0.065e | 0.762±0.074b | 0.74±0.056b | 0.585±0.051c |
| *P* | W | 0.133 | 0 | 0.011 | 0 | 0.016 | 0.012 |
|  | N | 0.297 | 0 | 0 | 0.003 | 0.002 | 0.022 |
|  | W*N | 0.004 | 0.001 | 0 | 0 | 0 | 0 |
| K(mg/g) | N0W80 | 6.285±0.392bc | 7.156±0.506a | 7.752±0.536b | 4.973±0.107d | 5.638±0.159ab | 4.353±0.255a |
|  | N0W50 | 6.492±0.533bc | 6.852±0.257ab | 10.361±0.271a | 6.608±0.341a | 5.853±0.314a | 4.184±0.174ab |
|  | N0W20 | 7.22±0.208a | 6.359±0.227b | 9.622±0.798a | 5.228±0.504cd | 5.613±0.404ab | 4.08±0.047b |
|  | N150W80 | 6.26±0.073c | 6.735±0.738ab | 9.994±0.576a | 5.244±0.308cd | 5.219±0.318b | 4.017±0.129b |
|  | N150W50 | 6.788±0.122ab | 7.343±0.342a | 9.806±0.631a | 5.587±0.153bc | 4.617±0.287c | 3.971±0.167b |
|  | N150W20 | 7.217±0.352a | 6.728±0.229ab | 9.53±0.268a | 5.801±0.408b | 5.568±0.321ab | 4.123±0.094ab |
| *P* | W | 0 | 0.048 | 0.001 | 0 | 0.098 | 0.383 |
|  | N | 0.505 | 0.411 | 0.029 | 0.667 | 0 | 0.018 |
|  | W*N | 0.552 | 0.094 | 0 | 0 | 0.004 | 0.076 |
| Ca(mg/g) | N0W80 | 9.052±0.19a | 7.156±0.506a | 11.726±1.781ab | 6.423±0.301a | 11.298±0.34c | 13.551±1.277c |
|  | N0W50 | 8.563±0.671a | 6.852±0.257ab | 13.108±1.219ab | 6.507±0.592a | 15.325±1.145a | 16.542±0.457a |
|  | N0W20 | 9.313±0.539a | 6.359±0.227b | 12.523±1.794ab | 6.269±0.544a | 14.085±2.723ab | 16.29±0.879ab |
|  | N150W80 | 8.114±1.351a | 6.735±0.738ab | 13.8±0.567a | 6.07±0.357a | 13.968±1.653ab | 14.864±1.263bc |
|  | N150W50 | 8.266±1.156a | 7.343±0.342a | 13.083±1.253ab | 6.357±0.182a | 12.566±0.834bc | 15.74±0.917ab |
|  | N150W20 | 8.524±0.775a | 6.728±0.229ab | 11.06±0.636b | 4.899±0.247b | 11.826±1.186bc | 15.26±0.176ab |
| *P* | W | 0.511 | 0.002 | 0.144 | 0.001 | 0.221 | 0.001 |
|  | N | 0.074 | 0.045 | 0.718 | 0.001 | 0.22 | 0.65 |
|  | W*N | 0.747 | 0.01 | 0.044 | 0.015 | 0.003 | 0.038 |
| Mg(mg/g) | N0W80 | 7.023±0.439c | 6.717±0.413a | 4.265±0.119bc | 1.104±0.028c | 2.464±0.31a | 1.944±0.208b |
|  | N0W50 | 7.951±0.515ab | 4.918±0.261d | 4.883±0.261a | 1.469±0.182a | 3.368±0.938a | 2.114±0.146ab |
|  | N0W20 | 8.276±0.24ab | 6.126±0.332b | 4.085±0.512c | 1.284±0.075b | 3.397±0.923a | 2.228±0.053a |
|  | N150W80 | 7.672±0.285b | 6.621±0.305ab | 5.072±0.357a | 1.055±0.054c | 2.975±0.979a | 2.316±0.127a |
|  | N150W50 | 8.272±0.23ab | 4.602±0.222d | 4.719±0.354ab | 1.065±0.048c | 2.222±0.537a | 2.305±0.163a |
|  | N150W20 | 8.423±0.572a | 5.574±0.447c | 3.772±0.221c | 1.115±0.079c | 3.344±0.63a | 2.231±0.096a |
| *P* | W | 0 | 0 | 0 | 0.003 | 0.202 | 0.345 |
|  | N | 0.037 | 0.033 | 0.421 | 0 | 0.47 | 0.004 |
|  | W*N | 0.465 | 0.423 | 0.006 | 0.004 | 0.114 | 0.054 |
| Fe(mg/g) | N0W80 | 2.705±0.239d | 0.352±0.072a | 1.632±0.157a | 0.324±0.042ab | 0.33±0.068a | 0.315±0.038a |
|  | N0W50 | 4.021±0.363c | 0.359±0.049a | 1.13±0.137b | 0.363±0.027a | 0.318±0.077a | 0.274±0.026b |
|  | N0W20 | 3.135±0.295d | 0.427±0.092a | 1.037±0.123bc | 0.245±0.032c | 0.3±0.078ab | 0.254±0.013b |
|  | N150W80 | 3.93±0.504c | 0.403±0.074a | 1.732±0.339a | 0.336±0.029ab | 0.258±0.065ab | 0.342±0.011a |
|  | N150W50 | 4.646±0.135b | 0.468±0.129a | 0.941±0.068bc | 0.29±0.023bc | 0.181±0.073b | 0.25±0.029b |
|  | N150W20 | 5.32±0.467a | 0.425±0.095a | 0.826±0.072c | 0.243±0.033c | 0.235±0.083ab | 0.187±0.03c |
| *P* | W | 0 | 0.538 | 0 | 0.115 | 0.493 | 0 |
|  | N | 0 | 0.164 | 0.181 | 0 | 0.008 | 0.059 |
|  | W*N | 0.001 | 0.474 | 0.17 | 0.032 | 0.574 | 0.008 |
| Mn(ug/g) | N0W80 | 247.44±5.371bc | 0.352±0.072a | 65.323±6.275ab | 17.881±1.846a | 90.965±6.035ab | 80.604±7.286a |
|  | N0W50 | 245.075±4.803c | 0.359±0.049a | 56.66±4.836c | 16.763±0.858a | 102.781±7.151a | 81.048±6.74a |
|  | N0W20 | 261.428±5.318ab | 0.427±0.092a | 47.753±4.595d | 17.834±0.967a | 60.059±10.12c | 48.225±3.174c |
|  | N150W80 | 246.617±7.983c | 0.403±0.074a | 72.13±5.647a | 13.146±0.879b | 50.146±7.497c | 40.744±1.868c |
|  | N150W50 | 265.978±11.003a | 0.468±0.129a | 60.617±6.115bc | 17.913±2.191a | 53.212±10.826c | 78.051±9.175a |
|  | N150W20 | 259.138±14.777abc | 0.425±0.095a | 42.059±5.37d | 13.311±1.499b | 89.383±4.945b | 66.659±5.714b |
| *P* | W | 0.026 | 0.115 | 0 | 0.037 | 0.207 | 0 |
|  | N | 0.123 | 0.006 | 0.462 | 0 | 0 | 0.005 |
|  | W*N | 0.032 | 0 | 0.085 | 0.001 | 0 | 0 |
| Cu(ug/g) | N0W80 | 32.301±3.897a | 27.945±11.111a | 45.197±1.974a | 25.962±2.324c | 28.26±6.053b | 27.578±3.655c |
|  | N0W50 | 31.637±4.62ab | 35.42±13.342a | 43.76±3.725a | 35.489±2.296a | 36.23±2.662a | 28.387±3.317bc |
|  | N0W20 | 27.798±1.443ab | 30.91±13.113a | 47.025±6.821a | 35.163±2.656a | 13.784±1.887c | 32.293±4.703abc |
|  | N150W80 | 30.653±0.848ab | 38.644±11.959a | 41.736±5.338ab | 29.561±1.927b | 26.943±5.265b | 35.973±4.304a |
|  | N150W50 | 31.85±1.981ab | 40.408±20.783a | 41.418±2.292ab | 32.047±3.37ab | 29.365±4.039b | 35.407±4.424a |
|  | N150W20 | 27.599±1.996b | 26.407±11.686a | 35.531±1.268b | 32.116±1.548ab | 36.799±2.508a | 34.29±2.509ab |
| *P* | W | 0.016 | 0.437 | 0.568 | 0 | 0.005 | 0.693 |
|  | N | 0.64 | 0.524 | 0.003 | 0.343 | 0.008 | 0.002 |
|  | W*N | 0.787 | 0.561 | 0.075 | 0.015 | 0 | 0.25 |
| Zn(ug/g) | N0W80 | 32.301±3.897a | 35.76±7.63a | 58.431±3.493a | 17.585±1.063bc | 47.075±3.826ab | 124.928±8.781c |
|  | N0W50 | 31.637±4.62ab | 42±5.607a | 45.182±6.406b | 19.043±2.708ab | 53.803±7.731a | 150.765±8.012a |
|  | N0W20 | 27.798±1.443ab | 37.288±3.979a | 43.589±3.065b | 15.161±1.446c | 48.51±8.247ab | 141.274±14.075ab |
|  | N150W80 | 30.653±0.848ab | 39.195±3.085a | 43.089±3.394b | 19.824±2.021ab | 44.719±7.371ab | 117.2±7.558c |
|  | N150W50 | 31.85±1.981ab | 45.495±10.403a | 43.18±5.273b | 21.162±2.416a | 33.748±6.366c | 131.312±5.084bc |
|  | N150W20 | 27.599±1.996b | 39.603±5.782a | 42.357±6.565b | 21.091±1.797a | 41.07±4.634bc | 123.624±8.834c |
| *P* | W | 0.094 | 0.148 | 0.006 | 0.153 | 0.813 | 0.001 |
|  | N | 0.029 | 0.264 | 0.011 | 0.001 | 0.002 | 0.001 |
|  | W*N | 0.731 | 0.98 | 0.016 | 0.121 | 0.041 | 0.403 |
| B(ug/g) | N0W80 | 28.739±2.277d | 6.717±0.413a | 20.988±2.249a | 10.31±1.343a | 21.738±2.066a | 15.92±1.031a |
|  | N0W50 | 40.608±4.506bc | 4.918±0.261d | 16.108±2.29b | 9.844±1.425ab | 19.24±2.88ab | 15.019±1.511ab |
|  | N0W20 | 37.623±2.254bcd | 6.126±0.332b | 15.109±2.038bc | 7.907±1.18c | 20.39±0.945a | 16.139±0.731a |
|  | N150W80 | 31.392±3.991cd | 6.621±0.305ab | 20.217±2.554a | 8.408±0.709bc | 17.154±1.11bc | 13.855±1.74bc |
|  | N150W50 | 46.2±10.127b | 4.602±0.222d | 13.382±1.318bc | 7.301±0.8c | 15.69±2.103c | 12.262±0.855c |
|  | N150W20 | 57.144±8.01a | 5.574±0.447c | 12.188±1.293c | 8.253±0.63bc | 14.57±1.655c | 12.205±0.187c |
| *P* | W | 0 | 0 | 0 | 0.078 | 0.084 | 0.115 |
|  | N | 0.001 | 0 | 0.018 | 0.006 | 0 | 0 |
|  | W*N | 0.025 | 0 | 0.512 | 0.035 | 0.505 | 0.273 |

Note: Effects of N and water treatments on the plant and soil nutrient elements of *Quercus dentata*. Ammonia nitrogen (AN), Nitrate nitrogen (NN), available phosphorus (AP), Nitrogen (TN), Phosphorus (TP), Potassium (K), Calcium (Ca), Magnesium (Mg), Iron (Fe), Manganese (Mn), Copper (Cu), Zinc (Zn), and Boron (B) content. The different treatments represents the combinations of N (0 and 150kg·ha^−1^·yr^−1^) and W (80%, 50%, and 20% saturation soil moisture content). Values are mean ± standard deviation (n = 4). Statistically significant differences between treatments are indicated by lowercase letters, as determined by Duncan's post-hoc test (*P* ≤ 0.05). General liner model was applied to examine the individual and interactive effects of N and water on different parameters.

**Table S2** Component factors of principle component analysis of grading indices

| Index | Component | | | | | | | |
| --- | --- | --- | --- | --- | --- | --- | --- | --- |
|  | 1 | 2 | 3 | 4 | 5 | 6 | 7 | 8 |
| height | -0.155 | 0.455 | **0.669** | 0.114 | 0.262 | 0.046 | -0.08 | 0.211 |
| diameter | 0.403 | 0.208 | 0.352 | 0.211 | 0.573 | -0.391 | -0.061 | 0.031 |
| root-shoot ratio | -0.386 | 0.3 | -0.373 | 0.31 | 0.267 | 0.212 | 0.339 | 0.278 |
| total biomass | -0.24 | **0.655** | -0.588 | -0.036 | -0.157 | -0.09 | -0.197 | 0.135 |
| root biomass | -0.271 | **0.668** | **-0.609** | 0.003 | -0.115 | -0.05 | -0.133 | 0.171 |
| chlorophyll content | -0.502 | 0.007 | 0.403 | -0.325 | 0.445 | -0.009 | 0.039 | -0.157 |
| SC | 0.697 | 0.402 | -0.341 | -0.092 | 0.363 | -0.206 | 0.073 | 0.116 |
| Ure | **0.824** | 0.077 | -0.112 | -0.219 | 0.359 | -0.211 | -0.073 | 0.041 |
| NN | 0.298 | **0.683** | 0.428 | 0.124 | 0.089 | 0.322 | -0.268 | -0.078 |
| AN | -0.068 | -0.124 | 0.546 | -0.363 | -0.241 | 0.413 | 0.363 | 0.197 |
| AP | 0.752 | -0.217 | -0.023 | 0.464 | -0.179 | 0.094 | 0.089 | -0.182 |
| TN | -0.05 | 0.475 | 0.405 | 0.098 | -0.453 | -0.499 | 0.301 | 0.013 |
| TP | -0.387 | 0.388 | -0.316 | 0.688 | -0.085 | -0.068 | 0.151 | -0.159 |
| K | **0.872** | -0.011 | 0.013 | 0.07 | -0.001 | 0.178 | 0.226 | 0.138 |
| Ca | 0.043 | -0.517 | -0.544 | 0.107 | 0.139 | 0.453 | -0.228 | 0.262 |
| Mg | 0.786 | 0.367 | 0.097 | 0.152 | -0.208 | 0.013 | 0.293 | 0.13 |
| B | 0.681 | 0.386 | 0.209 | -0.185 | -0.268 | 0.039 | -0.243 | 0.043 |
| Zn | 0.389 | **0.603** | -0.177 | -0.208 | 0.051 | 0.355 | 0.121 | -0.438 |
| Fe | 0.438 | 0.589 | 0.308 | 0.044 | -0.27 | 0.226 | -0.409 | 0.059 |
| Cu | -0.554 | 0.291 | -0.349 | -0.419 | -0.088 | -0.075 | -0.005 | -0.471 |
| Mn | 0.585 | 0.424 | -0.278 | -0.201 | 0.26 | 0.245 | 0.379 | -0.156 |
| *P*_n_ | -0.61 | 0.35 | 0.271 | 0.342 | 0.263 | 0.208 | -0.042 | -0.112 |
| *G*_s_ | **-0.892** | 0.362 | 0.031 | -0.094 | 0.099 | 0.099 | 0.088 | 0.152 |
| *C*_i_ | -0.688 | 0.445 | 0.001 | -0.39 | -0.069 | -0.051 | 0.11 | 0.344 |
| *T*_r_ | **-0.83** | 0.038 | 0.29 | 0.325 | 0.006 | 0.142 | 0.018 | -0.172 |

Note: Principal Component Analysis (PCA) illustrating the effects of N and water treatments on the growth and soil parameters of *Quercus dentata*. Plant growth indices include Height increment, Stem diameter, Root-shoot ratio, Root biomass, Total biomass, Chlorophyll content, Net photosynthetic rate (*P*_n_), Stomatal conductance (*G*_s_), Intercellular CO_2_ concentration (*C*_i_), and Transpiration rate (*T*_r_). Soil physicochemical properties include Ammonia nitrogen (AN), Nitrate nitrogen (NN), available phosphorus (AP), Total nitrogen (TN), Total phosphorus (TP), Potassium (K), Calcium (Ca), Magnesium (Mg), Iron (Fe), Manganese (Mn), Copper (Cu), Zinc (Zn), Boron (B), Urease (Ure), and Sucrase (SC). The bolded values represent the dominant loadings of each variable (index) in relation to the respective principal components (PCs).

**Table S3** Factor eigenvalues and contribution rate of grading indices

| Component | Initial eigenvalue | | |
| --- | --- | --- | --- |
|  | Total | Variance % | Cumulative % |
| 1 | 7.862 | 31.448 | 31.448 |
| 2 | 4.265 | 17.059 | 48.507 |
| 3 | 3.298 | 13.191 | 61.698 |
| 4 | 1.882 | 7.529 | 69.227 |
| 5 | 1.645 | 6.579 | 75.805 |
| 6 | 1.392 | 5.568 | 81.373 |
| 7 | 1.114 | 4.455 | 85.828 |
| 8 | 1.045 | 4.18 | 90.008 |

**Table S4** Permutation test for RDA under reduced model

| Component | ANOVA | | |
| --- | --- | --- | --- |
|  | Variance | *F* | *P* |
| SC | 0.248 | 1.15 | 0.341 |
| Ure | 0.17 | 0.787 | 0.539 |
| NN | 0.297 | 1.378 | 0.226 |
| AN | 0.2 | 0.926 | 0.456 |
| AP | 0.57 | 2.638 | 0.046 |
| TN | 0.127 | 0.589 | 0.729 |
| TP | 0.213 | 0.987 | 0.407 |
| K | 0.218 | 1.012 | 0.411 |
| Ca | 0.272 | 1.258 | 0.315 |
| Mg | 0.073 | 0.337 | 0.922 |
| B | 0.106 | 0.489 | 0.819 |
| Zn | 0.116 | 0.536 | 0.774 |
| Fe | 0.222 | 1.026 | 0.39 |
| Cu | 0.267 | 1.236 | 0.298 |
| Mn | 0.32 | 1.484 | 0.205 |

Note: Distribution pattern of plant growth indices and physicochemical parameters by redundancy analysis (RDA). Plant growth indices include Height increment, Stem diameter, Root-shoot ratio, Root biomass, Total biomass, Chlorophyll content, Net photosynthetic rate (*P*_n_), Stomatal conductance (*G*_s_), Intercellular CO_2_ concentration (*C*_i_), and Transpiration rate (*T*_r_). Soil physicochemical properties include Ammonia nitrogen (AN), Nitrate nitrogen (NN), available phosphorus (AP), Total nitrogen (TN), Total phosphorus (TP), Potassium (K), Calcium (Ca), Magnesium (Mg), Iron (Fe), Manganese (Mn), Copper (Cu), Zinc (Zn), Boron (B), Urease (Ure), and Sucrase (SC).
